# Smad4 Feedback Enhances BMPR1B Transcription in Ovine Granulosa Cells

**DOI:** 10.3390/ijms20112732

**Published:** 2019-06-04

**Authors:** Anwar Abdurahman, Xing Du, Yilong Yao, Yiming Sulaiman, Jueken Aniwashi, Qifa Li

**Affiliations:** 1 College of Animal Science and Technology, Nanjing Agricultural University, Nanjing 210095, China; 2016205001@njau.edu.cn (A.A.); duxing@njau.edu.cn (X.D.); m18751967328@163.com (Y.Y.); 2 College of Animal Science and Technology, Xinjiang Agricultural University, Urumqi 830001, China; ysulaiman@xjau.edu.cn (Y.S.); Jueken62@163.com (J.A.)

**Keywords:** sheep, *BMPR1B*, transcription factor, Smad4, granulosa cell apoptosis

## Abstract

BMPR1B is a type 1B receptor of the canonical bone morphogenetic protein (BMP)/Sma- and mad-related protein (Smad) signaling pathway and is well known as the first major gene associated with sheep prolificacy. However, little is known about the transcriptional regulation of the ovine *BMPR1B* gene. In this study, we identified the ovine *BMPR1B* gene promoter and demonstrated that its transcription was regulated by Smad4. In sheep ovarian follicles, three transcriptional variants of *BMPR1B* gene with distinct transcription start sites were identified using 5′ RACE assay while variants II and III were more strongly expressed. Luciferase assay showed that the region −405 to −200 nt is the PII promoter region of variant II. Interestingly, two putative Smad4-binding elements (SBEs) were detected in this region. Luciferase and ChIP assay revealed that Smad4 enhances PII promoter activity of the ovine *BMPR1B* gene by directly interacting with SBE1 motif. Furthermore, in the ovine granulosa cells, Smad4 regulated BMPRIB expression, and BMPRIB-mediated granulosa cells apoptosis. Overall, our findings not only characterized the 5’ regulatory region of the ovine *BMPR1B* gene, but also uncovered a feedback regulatory mechanism of the canonical BMP/Smad signaling pathway and provided an insight into the transcriptional regulation of *BMPR1B* gene and sheep prolificacy.

## 1. Introduction

The transformation growth factor-β (TGF-β) cytokine superfamily is one of the protein families with the largest number of members (more than 33 proteins) exhibiting the most diverse functions in organisms [1,2]. Based on sequence homology, receptor subtypes, and signaling specificity, this superfamily could be further classified into several subfamilies, such as bone morphogenetic protein (BMP), TGF-β, and activin [1]. Members of BMP subfamily exhibit similar sequences and the same BMP type receptor, and act via a common downstream signaling pathway, BMP signaling pathway [3]. Unlike the TGF-β signaling pathway, in which the ligands (including TGF-β1, TGF-β2, and TGF-β3) first bind to type II receptors (TGFBR2), BMPs bind strongly to the type I receptors (BMPR1A and BMPR1B) alone and only weakly to type II receptor, BMPR2 [2,4]. BMPR activation induces Smad1, Smad5, and Smad8, via a phosphorylation mechanism, and then, SMAD1/5/8 forms an oligomeric complex with Smad4. Finally, this complex enters the nucleus to control transcription of target genes, and thus, acts as a specific transcriptional effector and influences various biological functions [5,6]. 

The BMP/Smad signaling pathway is known to be involved in follicular development, ovulation, and female reproduction, especially fecundity in domestic animals. In pigs, multiple core members of this signaling pathway, such as *BMP5*, *BMP6*, *BMP7*, *BMP15*, and *BMPR1B*, have been identified as candidate genes responsible for reproductive traits through genome-wide association analysis (GWAS) and whole-genome sequencing (WGS) [7,8,9]. In sheep, three (*BMP15*, *BMPR1B*, and *GDF9*) of the four major genes (*BMP15*, *BMPR1B*, *GDF9*, and *B4GALNT2*) identified to affect the high fecundity of sheep are core components of the BMP/Smad signaling pathway [10,11]. *BMPR1B* is the first major gene identified for ovine high fecundity traits, including ovulation rate (OR) and litter size (LS), and A746G variant (also known as FecB loci) is the only major effector mutation found in *BMPR1B* gene at present [12]. Mechanically, the ewes carrying *FecB* gene have higher LS and OR, fewer apoptotic granulosa cells, and higher ovarian BMPR1B levels [13,14]. A more recent report showed that BMPR1B is an important anti-apoptotic cytokine found in ovine granulosa cells [12].

Our previous report showed that the expressions of core components, including *BMPR1B*, of the canonical BMP/Smad signaling pathway, were significantly different between ovaries of high- and low-fecundity Hu sheep [6]. However, little is known about the transcriptional regulation of the ovine *BMPR1B* gene. In this study, we identified and characterized the transcription start sites and the core promoter of the ovine *BMPR1B* gene, and investigated the transcriptional regulation of its 5′ regulatory region. 

## 2. Results

### 2.1. Identification of the Transcription Start Sites of the Ovine BMPR1B Gene

**Figure 1:**
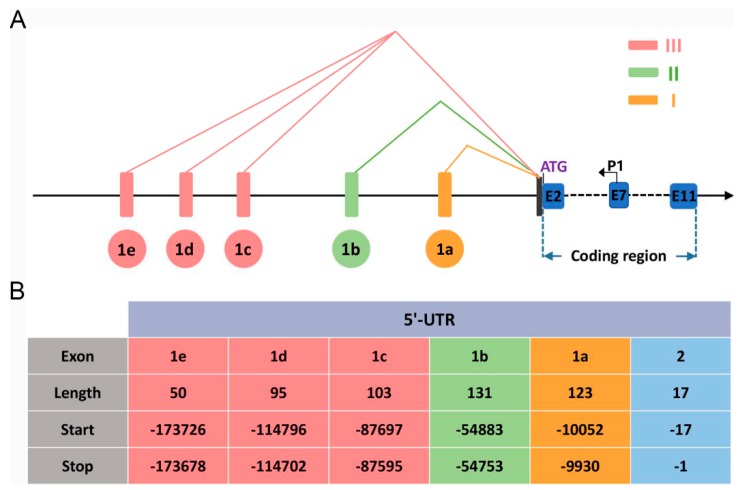
Identification of the transcription start sites of the ovine *BMPR1B* gene. (**A**) Schematic diagram showing the different 5′-UTRs of the ovine *BMPR1B* gene. Exons in the 5′-UTR are shown as long boxes and each transcript is represented by polylines. Exons in the coding region are shown as blue boxes. The translation start site is ATG. P1 is the primer used for 5′-RACE assay. (**B**) Characteristics of exons on the 5′-UTR of the ovine *BMPR1B* gene. Nucleotide numbering is relative to +1 at the initiating ATG codon.

To understand the characterization of the ovine *BMPR1B* gene 5′ regulatory region, we first identified the transcription start sites of the ovine *BMPR1B* gene using 5′ RACE assay. The first-strand was obtained using cDNA from follicles of Hu sheep ovaries as a template. Nested PCR was performed using first-stand as a template, and P1 as specific primers. Interestingly, clone sequencing showed that three sequences with different nucleotide sequences were identified within the amplification fragment (Figure 1A), suggesting that the ovine *BMPR1B* gene may have at least three transcript variants of 5′-UTR or three transcription start sites. 

BLAST with sheep genome sequence showed that these transcript variants consist of a partial sequence of exon containing the translation start codon ATG, and five novel short exons upstream of this exon, designated as exon 1a–1e, and the exon containing ATG was defined as exon2 (Figure 1B). The 5′-UTR of transcript variant I was composed of exon 1a (123 bp) and a part of exon 2 (17 bp), variant II included exon 1b (131 bp) and a part of exon 2 (17 bp), while variant III consisted of exon 1c (103 bp), 1d (95 bp), 1e (50 bp), and a part of exon 2 (17 bp). The full length of the 5′-UTRs of the three transcript variants were 140 bp, 148 bp, and 265 bp, respectively (Figure S1).

### 2.2. Identification of PII Promoter Region of the Ovine BMPR1B Gene

**Figure 2:**
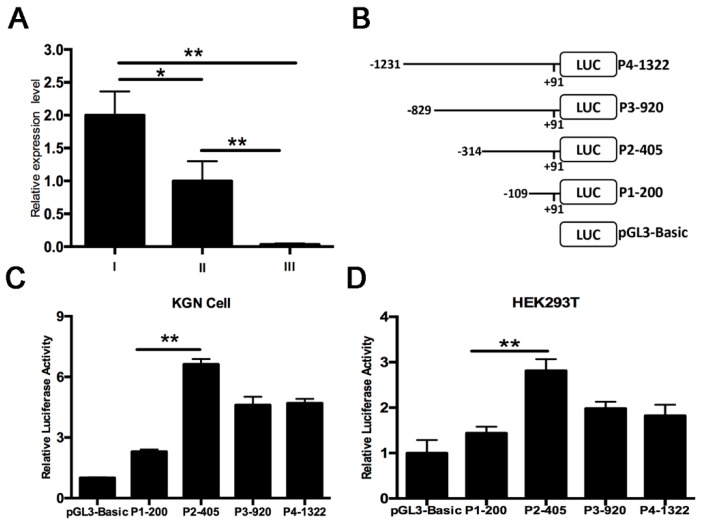
Identification of PII core promoter region of the ovine *BMPR1B* gene. (**A**) mRNA levels of the transcript variants of the ovine *BMPR1B* 5′-UTR in follicles of sheep ovary. (**B**) The schematic diagram of the deletion constructs. The 5′-end of transcript variant II was defined as +1. (**C**,**D**) Luciferase assay. The deletion constructs were transfected into KGN cells (**C**) and HEK293T cells (**D**), and luciferase activity was measured using a Dual-Luciferase Reporter Assay System. Bars represent the mean ± SEM of at least three repeats. * *p* < 0.05, ** *p* < 0.01.

qRT-PCR showed that transcript variants I and II were strongly expressed in ovarian follicles of Hu sheep (Figure 2A), indicating that transcript variants I and II were main variants located in the ovine ovary. To further explore transcriptional regulation of the ovine *BMPR1B* gene, we first identified the promoter region of the transcript variant II, one of the main variants. Four deletion fragments (–109/+91, –314/+91, –829/+91 and –1231/+91, the 5′-end of transcript variant II was defined as +1) were isolated from the 5′ regulatory region of transcript variant II, and then, inserted into luciferase reporter vector pGL3-basic. Subsequently four deletion constructs were successfully generated, and termed as P1-200, P2-405, P3-921, and P4-1322 (Figure 2B). Deletion constructs were transfected into KGN cells (a granulosa cell line) and HEK293T cells, and dual-luciferase reporter assay showed that luciferase activity of these four deletion constructs was significantly higher than that of the control group and significantly higher luciferase activity in plasmid P2-405-treated KGN cells compared to the other three plasmid-treated KGN cells (Figure 2C). Consistent with this, we also observed similar results in HEK293T cells (Figure 2D). Taken together, these data suggested that a 205 bp DNA region from −405 to −200 nt was the promoter region of transcript variant II, which was termed as PII core promoter of the ovine *BMPR1B* gene.

### 2.3. Characterization of the PII Core Promoter Region of the Ovine BMPR1B Gene

**Figure 3:**
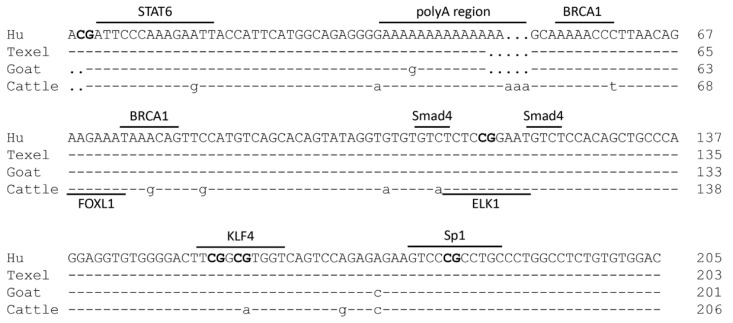
Sequence and putative regulatory elements of the PII core promoter region of the ovine *BMPR1B* gene. Underline indicates potential binding sites for transcription factors and polyA region. Bold letters represent CG site. Dot represents base deletion.

The PII core promoter of Hu sheep *BMPR1B* gene consists of 63 A (30.73%), 40 T (19.51%), 49 C (23.90%), and 53 G (25.85%) bases (Figure 3). The nucleotide sequence of the PII core promoter region is 99.02% identical with that of Texel sheep; the polyA region, extending from −276 to −265 nt, consists of 12 A bases in Texel, with 14 A bases in Hu sheep. Besides, PII core promoter is 97.07% and 92.79% identical with goat (*Capra hircus*) and cattle (*Bos taurus*) *BMPR1B* gene, respectively. The multiple putative binding sites for transcription factors, such as STAT6 (Signal transducer and activator of transcription 6), BRCA1 (Breast cancer 1), FOXL1 (Forkhead box protein L1), Smad4 (Sma-and mad-related protein 4), ELK1 (Ets-like protein-1), KLF4 (Kruppel-like factor 4), and Sp1 (Specificity protein 1) were determined in the PII core promoter region of the ovine *BMPR1B* gene. They were predicted using an online tool with a relative score of more than 82.0 (Figure 3), indicating that any one or more of these transcription factors may be involved in transcriptional regulation of the ovine *BMPR1B* gene and furthermore, the sequence of binding sites for most of the transcription factors among different species in *Bovinae*. In addition, CpG island was not predicted in the 5′ regulatory region of the transcript variant II; however, five CG loci were found in the PII core promoter region, suggesting that ovine *BMPR1B* gene may be regulated by methylation. 

### 2.4. Smad4 Enhances PII Promoter Activity of the Ovine BMPR1B Gene

**Figure 4:**
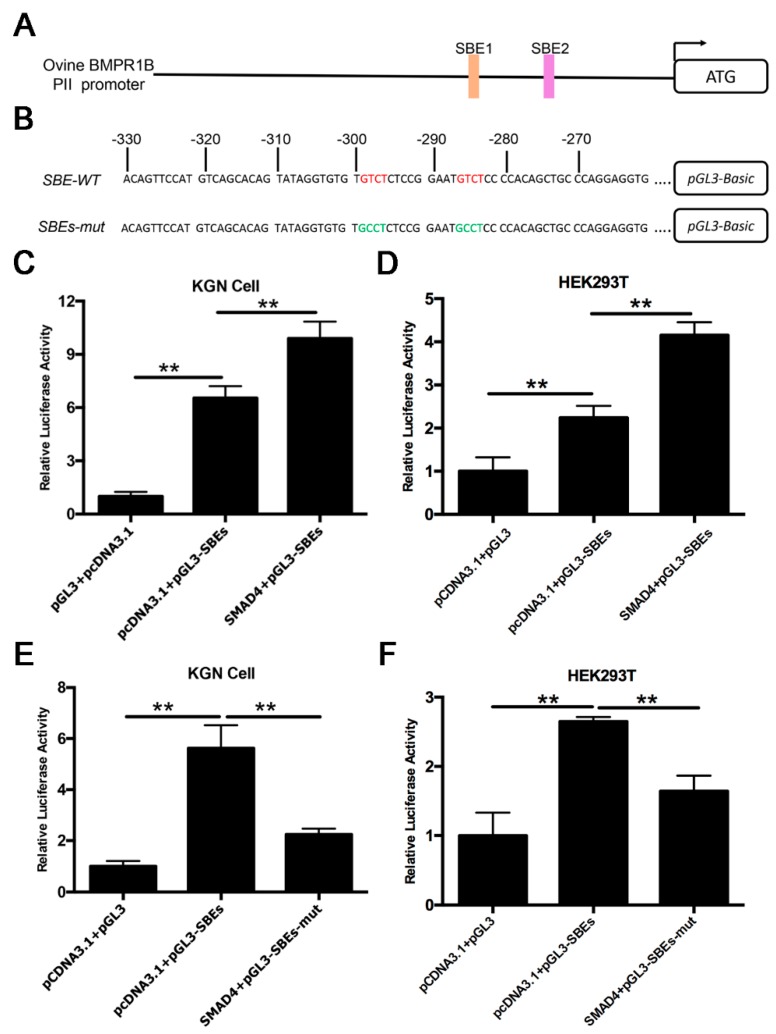
Smad4 enhances transcription activity of the ovine *BMPR1B* gene. (**A**) The SBE motifs were located in the PII promoter of the ovine *BMPR1B* gene. Two SBE motifs were detected at −299/−296 and −286/−283 nt in the PII core promoter. The 5′-end of transcript variant II was defined as +1. (**B**) The luciferase reporter constructs of the PII core promoter. PII core promoter region was isolated and subcloned into luciferase reporter vector pGL3-basic. Red letters indicate SBE motif. Green letter indicate mutated SBE motif. (**C**,**D**) Smad4 increases PII promoter activity. PII promoter reporter construct was co-transfected with pcDNA3.1-Smad4 into KGN cells (**C**) and HEK293T cells (**D**), luciferase activity was detected using a Dual-Luciferase Reporter Assay System. (**E**,**F**) Smad4 had no effect on activity of SBEs mutant-type PII promoter in KGN cells (**E**) and HEK293T cells (**F**). Bars represent the mean ± SEM of at least three repeats. ** *p* < 0.01.

Smad4 is the core component of the BMP/Smad signaling pathway, and usually acts as a transcription factor to positively or negatively regulate target genes by direct binding to Smad4-binding sites (SBEs) of the target promoter [11,15]. Notably, two SBE motifs (GTCT) were discovered on the PII core promoter region of the ovine BMPR1B gene (Figure 4A). To investigate whether transcription factor, Smad4, regulates PII core promoter activity of the ovine *BMPR1B* gene, we generated the luciferase reporter vector of PII core promoter (Figure 4B), and co-transfected it with pcDNA3.1-Smad4, the overexpression vector of Smad4, into KGN and HEK293T cells. A luciferase assay revealed that overexpression of Smad4 significantly increased PII core promoter activity of the ovine BMPR1B gene in both KGN and HEK293T cells (Figure 4C,D); however, the activity of SBEs mutant-type PII core promoter remained unaffected (Figure 4E,F). These data suggested that Smad4 enhances PII promoter activity of the ovine *BMPR1B* gene.

### 2.5. Smad4 Direct Binds to SBE Motif of the Ovine BMPR1B Promoter Region

**Figure 5:**
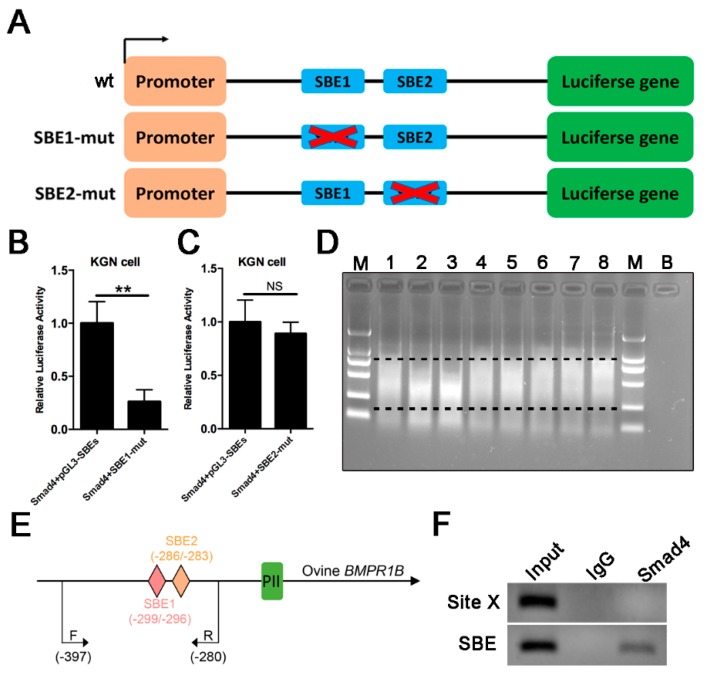
Smad4 directly binds to SBE motifs of the ovine *BMPR1B* promoter region. (**A**) The luciferase reporter construct of individual SBE motif-mutant type PII promoter. (**B**,**C**) Luciferase assay. SBE1 motif-mutant type (**B**) or SBE2 motif-mutant type (**C**) PII promoter reporter construct was co-transfected with pcDNA3.1-Smad4 into KGN cells, and luciferase activity was detected using a Dual-Luciferase Reporter Assay System. (**D**) Ultrasonic time optimization. (**E**) The location of primers used for ChIP assay. F indicates forward primer. R indicates reverse primer. (**F**) ChIP assay. Bars represent the mean ± SEM of at least three repeats. ** *p* < 0.01. ns, no significant.

To investigate which SBE motifs are involved in Smad4 regulation of PII promoter of the ovine *BMPR1B* gene, we constructed the luciferase reporter vector of the individual SBE motif-mutant type PII promoter (Figure 5A), and co-transfected with pcDNA3.1-Smad4 into cells. Luciferase assay showed that overexpression of Smad4 had no effect on luciferase activity of the SBE1 mutant-type PII promoter (Figure 5B), while SBE2 mutant did not influence Smad4-mediated regulation of PII promoter activity (Figure 5C), indicating that Smad4 increases PII promoter activity of the ovine *BMPR1B* gene via SBE1 motif within PII core promoter. To further investigate whether Smad4 interacts with the SBE1 motif of PII promoter in the ovine granulosa cells, Smad4-specific antibody was used for ChIP assay. The results are shown in Figure 5D–F, Smad4 can bind directly to SBE motif within the PII promoter of the ovine *BMPR1B* gene *in vivo*. Based on these results, we concluded that Smad4 increases PII promoter activity of the ovine *BMPR1B* gene by directly binding to SBE1 motif within the PII core promoter. 

### 2.6. Smad4 Positive Feedback Regulates BMPR1B Expression in the Ovine Granulosa Cells

**Figure 6:**
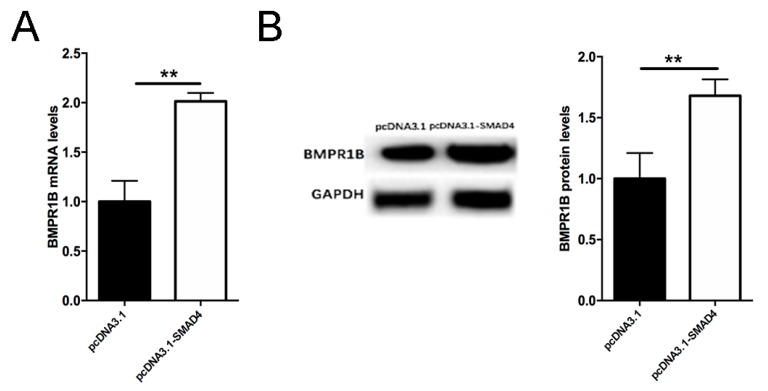
Smad4 induces endogenous BMPR1B expression in the ovine granulosa cells. Smad4 expression vector pcDNA3.1-Smad4 was transfected into the ovine granulosa cells cultured *in vitro*, and qRT-PCR and Western blotting was used to quantify mRNA (**A**) and protein (**B**) levels, respectively. Bars represent the mean ± SEM of at least three repeats. ** *p* < 0.01.

Smad4 is the final component of the canonical BMP/Smad signaling pathway. To further verify whether Smad4 exerts feedback regulatory effect on endogenous *BMPR1B* transcription, we performed a qRT-PCR assay to measure mRNA levels of *BMPR1B* in the Smad4-overexpessing ovine granulosa cells cultured *in vitro*. As excepted, overexpression of Smad4 significantly upregulated the mRNA levels of endogenous *BMPR1B* in the ovine granulosa cells (Figure 6A). Furthermore, Western blotting showed that protein levels of BMPR1B rise significantly in the ovine granulosa cells overexpressing Smad4 (Figure 6B). These data suggested that Smad4 is a transcriptional regulator of the ovine *BMPR1B* gene, which regulates BMPR1B expression in the ovine granulosa cells by positive feedback.

### 2.7. Smad4 Rescues BMPR1B-siRNA-Induced Cell Apoptosis in the Ovine Granulosa Cells

**Figure 7:**
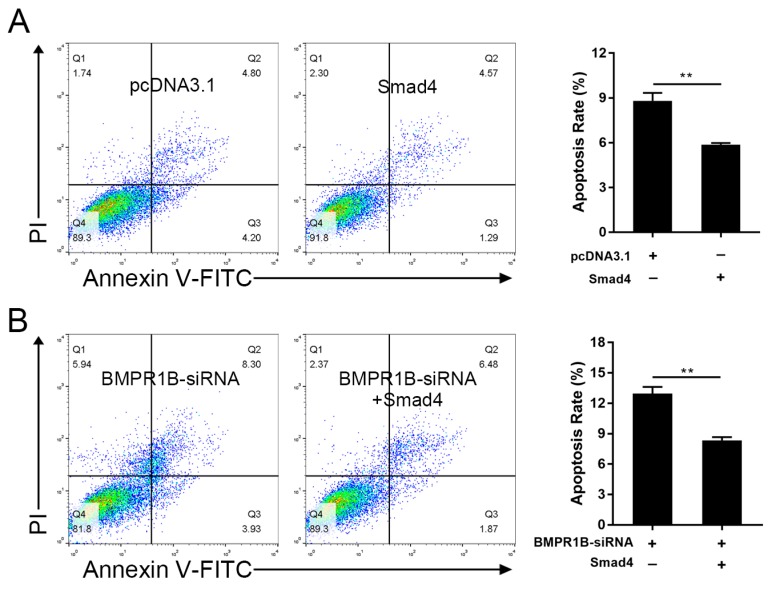
Smad4 regulates BMPR1B-mediated cell apoptosis in the ovine granulosa cells. (**A**) Smad4 suppresses granulosa cell apoptosis. Smad4 expression vector pcDNA3.1-Smad4 was transfected into the ovine granulosa cells cultured *in vitro*, and fluorescence-activated cell sorting (FACS) was performed to detect cell apoptosis rate. (**B**) Smad4 inhibits *BMPR1B*-siRNA-induced granulosa cell apoptosis. pcDNA3.1-Smad4 and *BMPR-1B*-siRNA were co-transfected into the ovine granulosa cells cultured *in vitro*, and FACS was performed to detect cell apoptosis rate. Bars represent the mean ± SEM of at least three repeats. ** *p* < 0.01.

BMPR1B was shown to be an inhibitor of apoptosis in the ovine granulosa cells [12]. To elucidate the role of its transcription factor, Smad4, in the ovine granulosa cell apoptosis, the cells were transfected with pcDNA3.1-Smad4, and their cell apoptosis was evaluated by a fluorescence-activated cell sorting (FACS) assay. As shown in Figure 7A, the cell apoptosis rate was significantly downregulated in the Smad4-overexpressing ovine granulosa cells. This result indicated that, similar to BMPR1B, Smad4 could also act as a negative regulator of apoptosis in the ovine granulosa cells. Next, we investigated whether BMPR1B mediates Smad4 regulation of the ovine granulosa cell apoptosis. Ovine granulosa cells were co-transfected with pcDNA3.1-Smad4 and *BMPR1B*-siRNA, and the results showed that overexpression of Smad4 significantly inhibited *BMPR1B*-siRNA-induced cell apoptosis in the ovine granulosa cells (Figure 7B). Taking these results together, we proved that Smad4 regulates BMPR1B-mediated cell functions in the ovine granulosa cells.

## 3. Discussion

*BMPR1B* is an important major gene associated with reproductive traits, including LS and OR, in sheep, and its major mutation, FecB, has been successful applied to breed the prolific strain of non-prolific breeds in multiple countries, as an effective genetic marker for marker-assisted selection (MAS) [10,16]. Unfortunately, the sequence of the regulatory region of the ovine *BMPR1B* gene remains unclear. Although Yao et al. [12] demonstrated that miR-125b regulates *BMPR1B* level by directly targeting its 3′-UTR, only the partial sequence of its 3′-UTR was isolated and characterized. Here, we identified, for the first time, the transcription start sites of the ovine *BMPR1B* gene, and elucidated the characteristics of its 5′-UTR, which might provide a foundation for further research on its transcriptional regulation.

Importantly, we also identified and characterized the PII core promoter, and a large number of transcription factor binding sites (TFBSs) were found in this region. Notably, some transcription factors, such as Sp1, BRCA1, and ELK1, are involved in female reproduction, similar to BMPR1B [17]. Sp1 belongs to the Sp/KLF family of transcription factors, which is essential for ovarian functions by regulating known female reproduction-related genes, such as *CYP11A1, CYP17*, *IRS-2*, and *RGS*, by direct binding to a consensus GC-rich motif within the promoter of these targets, in mammalian ovary [18,19,20,21]. *BRCA1* is known as a tumor suppressor gene for breast and ovarian cancer, and belongs to the family of ataxia-telangiectasia-mutated-mediated DNA double-strand break (DSB) repair genes and plays a critical role in homology-directed repair of DSBs [22]. BRCA1 is highly expressed in the ovary, and is associated with ovarian reserve, oocyte quality, and female fertility [23]. ELK-1 belongs to the ternary complex factor subfamily of Ets-domain transcription factors, which responds to extracellular signal-regulated kinase (ERK) activation, and controls ovarian granulosa cell functions, such as cell proliferation, migration, and steroid hormone secretion [17,24,25]. In addition, we also detected several CpG sites in the PII promoter region of the ovine *BMPR1B* gene, indicating that ovine *BMPR1B* transcription may be regulated by DNA methylation. A recent report showed that *BMPR1B* is one of the differentially methylated region-related genes in ovaries between high- and low-fecundity Hu sheep using deep whole-genome bisulfite sequencing (WGBS) [26].

Interestingly, we found that two SBE sites are located within the PII promoter of the ovine *BMPR1B* gene, and demonstrated that Smad4 controls *BMPR1B* transcription in the ovine granulosa cells, which acts as a transcription factor. In fact, binding with promoter regions of important functional genes and regulating their transcription are the main way for SMAD4 to exert its biological functions [11,27,28]. However, in mammalian granulosa cells, only a few key genes for controlling granulosa cell functions, have been identified as direct transcriptional targets of transcription factor SMAD4 [15,29]. For example, *CYP19A1*, a key gene in E2 production, has been shown to be directly regulated by SMAD4 at the transcriptional level, in granulosa cells [15]. In porcine granulosa cells, SMAD4 suppressed cell apoptosis through enhancing *miR-143*-targeted FSHR (a marker of granulosa cells) signaling pathway by direct binding to the promoter of *miR-143* gene [30]. In addition, several miRNAs associated with granulosa cell apoptosis and follicular atresia, including *miR-425* and *miR-1306*, are also direct transcriptional targets of SMAD4 in porcine granulosa cells [11,29]. In conclusion, our findings demonstrated, for the first time, that SMAD4 acting as a transcription factor, can directly bind to and regulate the core member of the canonical TGF-β/Smad signaling pathway.

SMAD4 is the final signal molecule of the canonical BMP/Smad signaling pathway. In this study, we showed that SMAD4 is a transcription activator of *BMPR1B*, an upstream signal molecule of SMAD4, in the ovine granulosa cells. This suggested that Smad4 can regulate the canonical BMP/Smad signaling pathway via positive feedback. The feedback regulatory mechanism has been proved to exist widely in the TGF-β family signaling pathway, especially in the canonical TGF-β/Smad signaling pathway [31]. SMAD2 and SMAD3, both are receptor-regulated Smads (R-SMADs), whose feedback regulates the canonical TGF-β/Smad signaling pathway, via *miR-520e*-TGFBR2 axis [32], interacting with the TMEPAI family, and the latter attenuates recruitment of Smad2/3 to the TGFBR1 [33,34]. Smad4, only the one common mediator Smad (Co-Smad) of the TGF-β family signaling pathway, has also been shown to be an important feedback modulator for the canonical TGF-β/Smad signaling pathway [11]. The enhancement of the canonical TGF-β/Smad signaling pathway by this positive feedback factor is usually mediated by several miRNA-TGFBR2 axes, such as *miR-425*-TGFBR2 axis [11], *miR-122-5p*-TGFBR2 axis [35], and *miR-1306*-TGFBR2 axis [29]. For the canonical BMP/Smad signaling pathway, *miR-17* family-BMPR2 axis and Noggin-BMP4 axis mediated feedback regulation of this positive feedback factor in the neuron [36] and in developing spinal cord [37], respectively. Therefore, our findings not only demonstrated, for the first time, that a feedback regulatory mechanism exists within the canonical BMP/Smad signaling pathway in ovarian granulosa cells, but also provided new evidence for feedback regulation within the BMP/Smad signaling pathway in mammals.

Functionally, we showed that Smad4 could rescue the granulosa cell apoptosis induced by BMPR1B silencing, demonstrating that this feedback regulation is involved in ovine granulosa cell apoptosis. It is well known that the canonical BMP/Smad signaling pathway is associated with fecundity in domestic animals, and is essential for gonadogenesis, steroidogenesis, follicular development, and ovulation [8,10,38]. Meanwhile, BMP/Smad signaling pathway also plays an important role in follicular atresia, another fate of follicular development [39,40,41]. Follicular atresia is triggered by apoptosis of granulosa cells, therefore, most of its core components of the canonical BMP/Smad signaling pathway are related to the follicular granulosa cell functions, especially granulosa cell apoptosis in mammals [11,42]. Ligands, BMP2 (in mice [43]), BMP4 and BMP7 (in cows [42]), and BMP15 (in humans [44]) have been shown to regulate apoptosis of granulosa cells. Two core receptors, BMPR1B and BMPR2, were found to regulate granulosa cell apoptosis in ovaries of humans [45] and sheep [12], respectively. For R-Smads, SMAD1 regulated COV434 cell (a human granulosa cell tumor-derived cell line) apoptosis [46], and SMAD5 suppressed human granulosa cell apoptosis via the FasL-Fas pathway [47,48], but SMAD8 has not been reported to regulate GC apoptosis. Only one Co-SMAD, SMAD4 has been widely demonstrated to be a strong regulator of granulosa cell apoptosis in the ovary of various mammals, such as rodents [46] and pigs [15,17], but not sheep. Furthermore, *in vivo*, both SMAD4 and BMPR1B have been shown to be strongly involved in follicular atresia of domestic animals including sheep [14], pigs [4], and yaks [41]. In conclusion, our study not only provided evidence for the canonical BMP/Smad signaling pathway-mediated regulation of granulosa cell apoptosis, filling in the gap of the role of SMAD4 in regulating granulosa cell apoptosis in sheep, but also confirmed, for the first time, that feedback regulation of this pathway is also involved in granulosa cell apoptosis, even follicular atresia of the ovine ovary.

In summary, we reported that ovine *BMPR1B* gene contains multiple transcriptional star sites, and several regulatory elements were identified within its PII core promoter region. We also showed that SMAD4 functions as a transcription factor, feedback induces *BMPR1B* transcription, and then, regulates BMPR1B-mediated cell apoptosis in the ovine granulosa cells. Overall, our findings suggested, for the first time, that feedback regulation exists within the canonical BMP/Smad signaling pathway in ovarian granulosa cells, and revealed that this feedback mechanism regulates granulosa cell apoptosis and provides a new insight into the mechanism of granulosa cell apoptosis, follicular development, and female reproduction in mammals.

## 4. Materials and Methods

### 4.1. Sample Preparation

Ear tissues and fresh ovary samples of Hu sheep were collected from Xilaiyuan sheep breeding farm (Taizhou, China). Ear tissues were stored in 75% ethyl alcohol, and used for DNA extraction. Ovary samples were kept in physiological saline, and used to isolate medium follicles and total RNA. Animal experiments were reviewed and approved by Nanjing Agricultural University Animal Care and Use Committee and performed in accordance with the Regulations for the Administration of Affairs Concerning Experimental Animals (China, Decree No.2 of the State Science and Technology Commission, approved on 14 November 1988, approval code: SYXK 2017-0027).

### 4.2. Cell Culture 

For the ovine granulosa cell culture, fresh ovaries were obtained from Hualing Slaughterhouse (Urumqi, China), immersed into sterilized saline at 37 °C, and then transported back to the laboratory within 2 h. Granulosa cells were isolated and cultured as previously described [11]. KGN and HEK293T cells were obtained from Nanjing built Biological (Nanjing, China) for Dual-Luciferases assay. KGN cells were seeded into T25 culture flasks and cultured in Roswell Park Memorial Institute 1640 medium (RPMI 1640) containing fetal bovine serum (10%, Atlanta Biologicals, Shanghai, China), penicillin G (100 U/mL), and streptomycin sulfate (100 μg/mL). HEK293T cells were cultured in Dulbecco’s Modified Eagle Medium (DMEM) containing fetal bovine serum (10%, Atlanta Biologicals), under similar conditions as KGN cells. 

### 4.3. Nucleic Acid Extraction and qRT-PCR

DNA was extracted using the conventional phenol–chloroform extraction method. TRIzol reagent (Invitrogen, Carlsbad, CA, USA) was used to isolate total RNA from the ovine ovarian follicles and granulosa cells. Then, cDNA was prepared using a Prime Script™ RT Master Mix (TaKaRa, Dalian, China). Quantitative RT-PCR (qRT-PCR) was carried out in triplicate using a kit (SYBR Premix Ex Taq, Takara), and *GAPDH* was used as an internal control. The 2^−ΔΔCT^ method was used to analyze relative levels of target genes. 

### 4.4. 5′ Rapid Amplification of cDNA Ends (RACE)

SMARTer^®^ RACE 5′/3′ Kits (TaKaRa) were used to obtain the 5′-UTR sequence of Hu sheep *BMPR1B* gene, according to the manufacturer’s instructions. Briefly, total RNA from follicles was reverse transcribed into cDNA using enzyme terminal deoxynucleotidyl transferase (TdT) which has the ability to add a string of identical nucleotides to the 5′ end of the cDNA, followed by PCR amplification with specific primers designed by *BMPR1B* reference sequence. PCR products were detected by gel electrophoresis and the purified fragments were cloned into pUC-19 vector. After sequencing and alignment, the full-length sequence of Hu sheep *BMPR1B* 5′ end was obtained. The primers used are listed in Table 1. 

### 4.5. Bioinformatics Analysis

NCBI database (https://www.ncbi.nlm.nih.gov/nuccore/) was used to obtain the ovine reference *BMPR1B* gene (NC_019463.2 for Texel sheep). The putative promoter region of Hu sheep *BMPR1B* gene was predicted using an online software Promoterscan (http://www-74bimas.cit.nih.gov/molbio/proscan/). The TFBSs were predicted using the online database Jaspar (http://jaspardev.genereg.net). 

### 4.6. Plasmid Construction

Smad4 expression vector, pcDNA3.1-Smad4, was prepared in our laboratory [14]. For identification of the core promoter of Hu sheep *BMPRIB* gene, we amplified the 5′ flanking sequence by deletion expression manner. The primers were designed as follows: SacI and XhoI endonuclease site was incorporated at the 5′ and 3′ end, respectively (Table 1). PCR products were purified using Axy prep^TM^ PCR cleanup kit (Axygen, Union city, CA, USA) and sub-cloned into the pGL3 luciferase reporter vector which was then transferred into *E. coli* to construct the luciferase reporter plasmids. The plasmids were extracted using an Endo-free plasmid mini kit II50 (Omega, Norcross, GA, USA), and all plasmids were validated by sequencing. TaKaRa MutanBEST Kit (TaKaRa) was used to generate SBE mutation-typed plasmids.

### 4.7. Luciferase Activity Assay

HEK293T and KGN cells were cultured into 12-well cell culture plates for 24 h. Transfection of reporter plasmids and measurement of luciferase activity were done as described in our previous report [11].

### 4.8. Western Blotting

Extraction and quantification of total protein in granulosa cells was performed using conventional methods and BCA Kit (Pierce, Shanghai, China). Western blotting was performed as described previously [11]. Primary antibodies were BMPR1B-specific antibody (Abcam, ab155058, Cambridge, UK, 1:1000) and GAPDH-specific antibody (Abcam, ab9482, 1:1000), and secondary antibody was anti-goat and anti-mouse IgG (1:1000; Origene, Herford, Germany). ImageJ software was used to analyze the bands.

### 4.9. Apoptosis Analysis

The apoptosis of granulosa cells in this study was determined using the Annexin V-FITC/propidium iodide apoptosis kit (KeyGene, Nanjing, China), according to the manufacturer’s protocol. Apoptosis rate of granulosa cells was calculated and analyzed using Flowjo software (v7.6, Stanford University, Stanford, CA, USA).

### 4.10. Chromatin Immunoprecipitation

Chromatin immunoprecipitation (ChIP) assay was performed using the SimpleChIP^®^ Plus Enzymatic Chromatin IP Kit (Cell Signaling Technology, MA, USA). The anti-SMAD4 antibody (sc-7154; Santa Cruz Biotechnology, CA, USA) was used as a specific antibody for ChIP assay, while IgG (Origene) was used as control. The primers for ChIP-PCR are listed in Table 1.

### 4.11. Statistical Analysis

Data were expressed as the mean ± S.E.M. of at least three independent experiments. Statistics analysis was performed using SPSS v20.0 software (SPSS Inc., Chicago, IL, USA). *p* <0.05 was considered as statistically significant.

## Figures and Tables

**Table 1:** The primer sequences.

Primer	Gene	Primer Sequence (5′-3′)	Size (bp)	Tm (°C)	Usage
P1	*BMPR1B*	GATTACGCCAAGCTTTTCGCCACGCCACTTTCCCATCC			RACE
P2	*BMPR1B*	F: GAGCTCAGCTGGGTCAGGCTCCTTTC R: CTCGAGAGACTCCTCTGCTGCCACTC	200	58	Deletion construction
P3	*BMPR1B*	F: GAGCTCACGATTCCCAAAGAATTACC R: CTCGAGAGACTCCTCTGCTGCCACTC	403	56	Deletion construction
P4	*BMPR1B*	F: GAGCTCTTCCCAGCATGAATCAGAGTC R: CTCGAGAGACTCCTCTGCTGCCACTC	921	57	Deletion construction
P5	*BMPR1B*	F: GAGCTCGGAACTGAGGATGTTGGATTG R: CTCGAGAGACTCCTCTGCTGCCACTC	1322	56	Deletion construction
P6	*BMPR1B*	F: AGCTCCCATGTCAGCACAGTATAGGTGTGTGCCTCTCCGG R: ACTGTGCTGACATGGGAGCT	298	56	Promoter construction
P7	*BMPR1B*	F: GTATAGGTGTGTGTCTCTCCGGAATGCCTCCACAGCTGCCCA R: CCGGAGAGACACACACCTATAC	298	56	Mutation construction
P8	*BMPR1B*	F: TGCTGGGCTCTTTAGTGG R: CTGGACAATGGTGGTGGC	237	60	qPCR for variant I
P9	*BMPR1B*	F: GGCAAGAAACAGGAGGCT R: CCACAGGCATCCCAGAGT	241	60	qPCR for variant II
P10	*BMPR1B*	F: CATAGACAACAGCCCACCAG R: GACCACAGGCATCCCAGA	264	60	qPCR for variant III
P11	*BMPR1B*	F: AGCACTCAAGGCAAACCA R: GGCCATGATGTAAGACTGAAAG	243	60	qPCR
P12	*GAPDH*	F: TGGAATGACATCTCGGTCTGGTA R: CACCATGGCTCAGAAGCACAC	247	58	qPCR
P13	*BMPR1B*	F: CCCAAAGAATTACCATTCATGGC R: GACATTCCGGAGAGACACACACCTA	118	63	ChIP-PCR

## Supplementary Materials

Supplementary materials can be found at https://www.mdpi.com/1422-0067/20/11/2732/s1.
