# Supplementary material for: Smad4 Feedback Enhances BMPR1B Transcription in Ovine Granulosa Cells
Source: Int J Mol Sci. 2019 Jun 4;20(11):2732. doi: 10.3390/ijms20112732 (PMC6600593; doi:10.3390/ijms20112732)
Supplement: Supplementary file 1 [file ijms-20-02732-s001.pdf]

Variant I 5'-UTR

```
1   CAGGATTAGA GCTGCCTACG GTGCTGGGCT CTTTAGTGGA CACCCCTGGC AGTTCCGTGC
61  ATCCTCCTCA TGTCTCATCC CAGGGAACCT GGCAACGATC TTACAAGCGT ACAGAAATCG
121 CGACAAACTT CCTTGATAAC
```

Variant II 5'-UTR

```
1   AGTTTGTAAC AGGCTTGTCT TTCACGGTCC TGTGTAGGGC TTGCTGGCAG GCTTGCCGGA
61  AGCTCTGACA AGAGTGGCAG CAGAGGAGTC TCCGTGAAAG AAGCAAGGCA AGAAACAGGA
121 GGCTTAAACA GCAAACTTCC TTGATAAC
```

Variant III 5'-UTR

```
1   TAAATCATCA AGGACGTTCT ACACTTTGGT TATCAGCAGC CTGTTTATCT GGTTCAAACT
61  TCTGCTGAAT CACAAGCATT TTCCGTTGAG CTATGACAAG AGAGGATACA AAAAGTTAAA
121 CAAGCAAGCC TGTCATACGT AGAAGACTCT AGATTGCTGC TGCTGCTAAG TCACTTCAGT
181 CATGTCCGAC TCTGTGTGAC CCCATAGACA ACAGCCCACC AGGCTCCCCC GTCCCTGGGA
241 TTCTCCAGCA AACTTCCTTG ATAAC
```

Figure S1. The sequences of the 5'-UTR of the ovine BMPR1B gene. Black letters indicate shared partial sequences of exon 2.
